# Case report: Target and immunotherapy of a lung adenocarcinoma with enteric differentiation, *EGFR* mutation, and high microsatellite instability

**DOI:** 10.3389/fimmu.2023.1266304

**Published:** 2024-01-25

**Authors:** Meiling Yang, Pengli Yu, Zhiyi He, Jingmin Deng

**Affiliations:** ^1^ Department of Pulmonary and Critical Care Medicine, The First Affiliated Hospital of Guangxi Medical University, Nanning, China; ^2^ Department of Medicine, Geneplus Beijing, Beijing, China

**Keywords:** pulmonary enteric adenocarcinoma, *EGFR*, MSI-H, immune checkpoint inhibitor, pMMR

## Abstract

**Background:**

Pulmonary enteric adenocarcinoma (PEAC) is a rare histological subtype of non-small-cell lung cancer (NSCLC) with a predominant (>50%) enteric differentiation component. The frequency of high microsatellite instability (MSI-H) is very low in lung cancer. EGFR tyrosine kinase inhibitors and immunotherapy are standard treatment for NSCLC patients, but their effectiveness in lung adenocarcinoma with pulmonary enteric differentiation is unknown.

**Case presentation:**

This report describes a 66-year-old man who was initially diagnosed with metastatic lung adenocarcinoma with *EGFR* mutation based on pleural fluid. A lung biopsy was obtained after 17 months of first-line icotinib treatment. Histological analysis of biopsy samples and endoscopic examination resulted in a diagnosis of adenocarcinoma with enteric differentiation. Next-generation sequencing of 1,021 genes showed *EGFR* E19del, T790M, and MSI-H, while immunohistochemical assay showed proficient expression of mismatch repair (MMR) proteins. Consequently, the patient was treated with osimertinib and had a progression-free survival (PFS) of 3 months. His treatment was changed to chemotherapy with/without bevacizumab for 6.5 months. Then, the patient was treated with one cycle of camrelizumab monotherapy and camrelizumab plus chemotherapy, respectively. The tumor continued to grow, and the patient suffered pneumonia, pulmonary fungal infections, and increased hemoptysis. He received gefitinib and everolimus and died 2 months later and had an overall survival of 30 months.

**Conclusion:**

In summary, our case describes a rare pulmonary enteric adenocarcinoma with an *EGFR*-activating mutation and MSI-H, responding to an EGFR tyrosine kinase inhibitor and poorly benefiting from an immune checkpoint inhibitor.

## Introduction

Pulmonary enteric adenocarcinoma (PEAC) is a rare variant of lung adenocarcinoma. According to the 2015 World Health Organization (WHO) classification, PEAC has been defined as primary pulmonary adenocarcinoma with more than 50% of intestinal differentiation components, and the tumor cells should be positive for at least one immunohistochemical marker of enteric differentiation, including CK20, CDX2, and MUC2 (1). The pathogenesis of PEAC and specific treatment plans have not been fully determined. At present, the treatment strategy for PEAC is similar to that of lung adenocarcinoma. The strategy methods derived from the literature for PEAC are mainly surgery and chemotherapy. Although several case reports have described immunotherapies in patients with PEAC, few have reported targeted therapy (2).

*KRAS* and DNA mismatch repair (MMR) genes are more frequently mutated in PEAC compared with those in other lung adenocarcinomas (3, 4). The high frequency of MMR mutation rates may facilitate the possibility of checkpoint-blocking immunotherapy for PEAC patients. The positive rate of *EGFR* mutations is approximately 16.7%, much lower than that of *KRAS* (3, 4). Few reports to date have described the effects of treatment with sequential *EGFR* tyrosine kinase inhibitors in PEAC. The present report describes the targets and immunotherapy of a lung adenocarcinoma with enteric differentiation, *EGFR*-activating mutation, and high microsatellite instability (MSI-H).

## Case report

**Figure 1:**
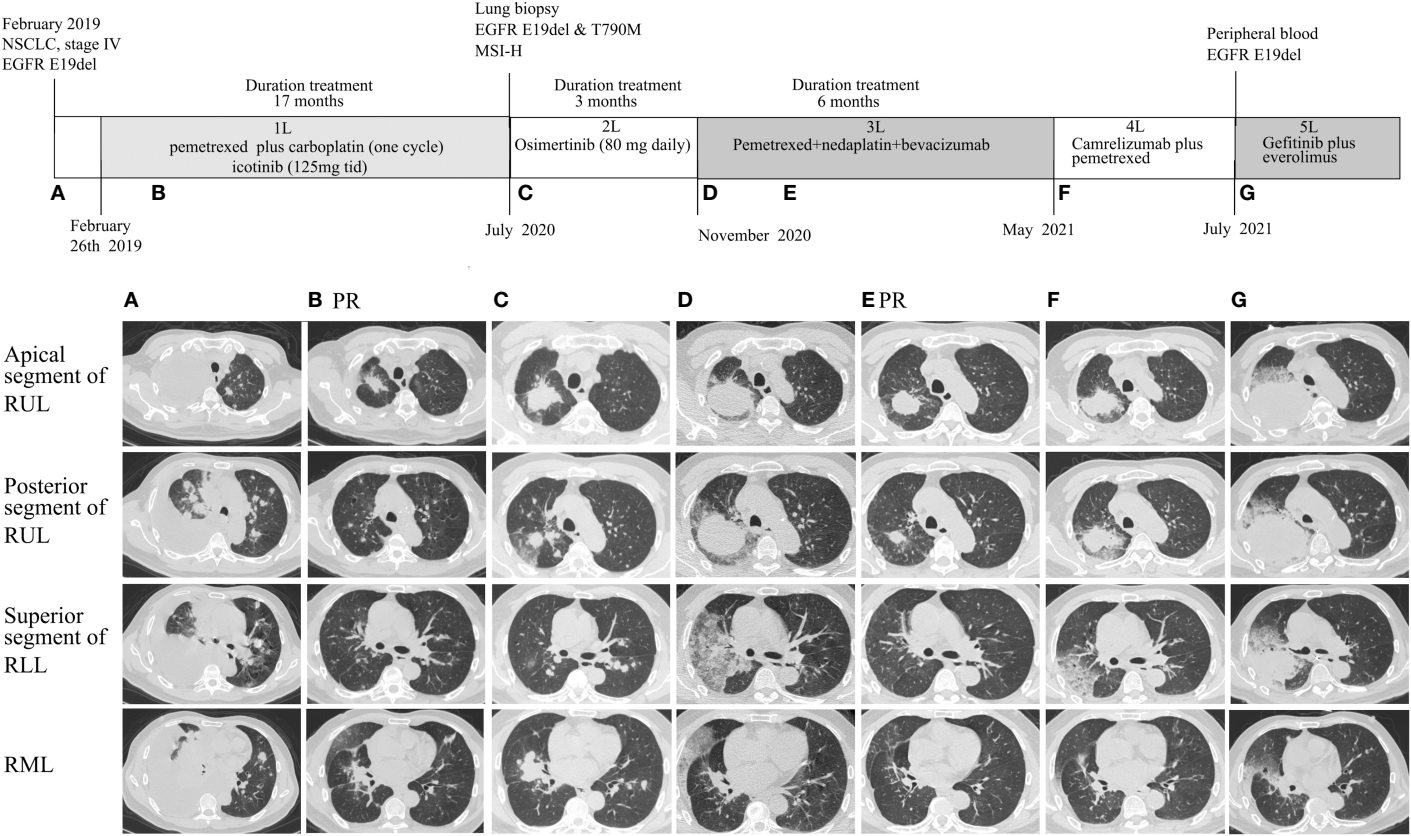
Radiographic evaluations of main lesions during the disease course. **(A)** Computed tomography (CT) scan at diagnosis. **(B)** CT scan showed lesions shrank after 1 month of first-line icotinib; **(C)** CT scan after resistance to icotinib. **(D)** CT scan after 3 months of osimertinib treatment; **(E)** CT scan before the second cycle of nedaplatin–pemetrexed–bevacizumab showing partial response; **(F)** CT after 6 months of third-line therapy showing disease progression; **(G)** CT scan after 2 months of immunotherapy. RUL, right upper lobe; RLL, right lower lobe; RML, right middle lobe; PR, partial response.

**Figure 2:**
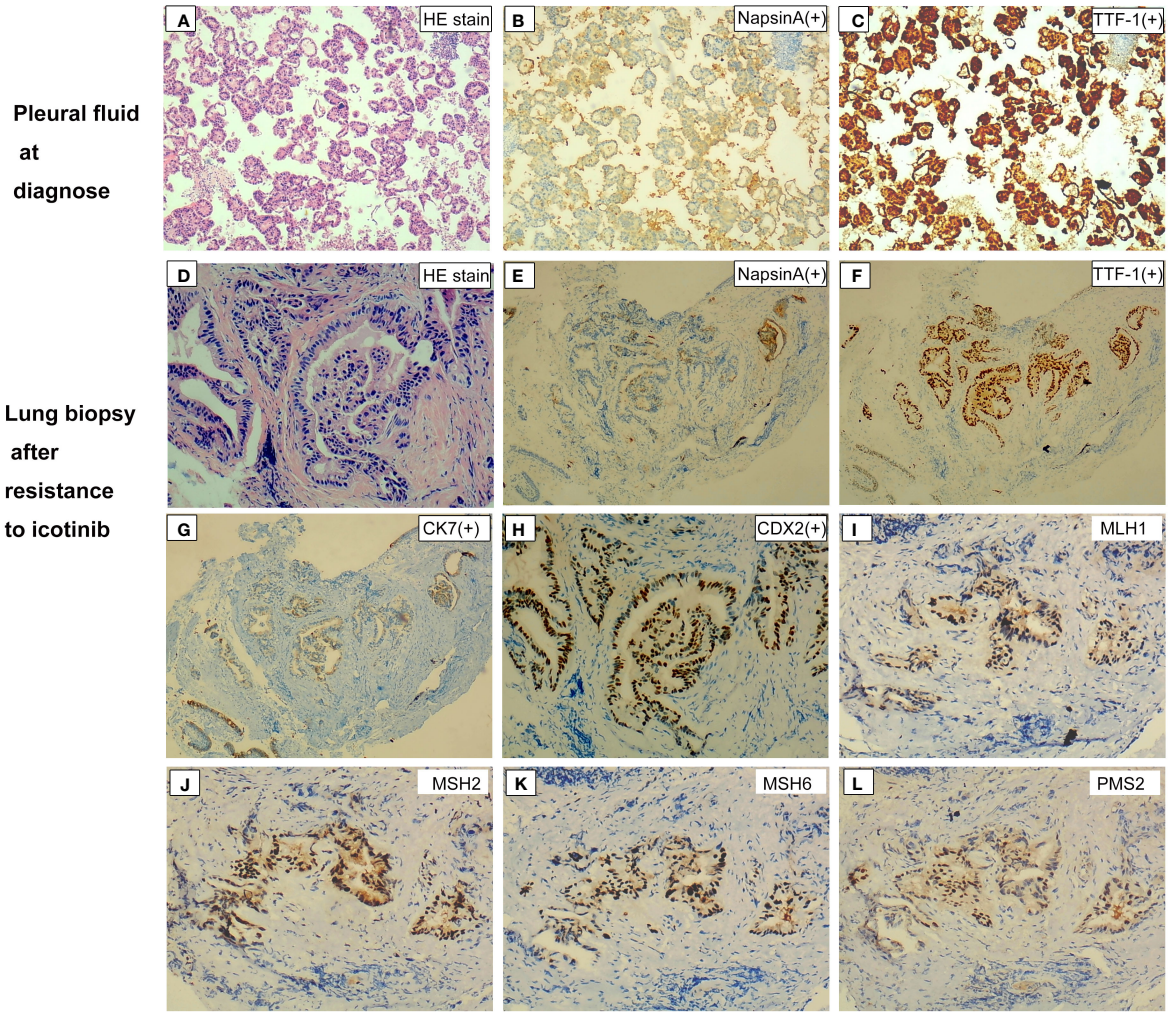
Hematoxylin and eosin staining and immunohistochemical findings of samples at different disease courses. **(A)** Circulating tumor cells in pleural fluid on hematoxylin and eosin staining at diagnosis. **(B, C)** Immunohistochemical staining was positive for NapsinA **(B)** and TTF-1 **(C)**. **(D)** Hematoxylin and eosin staining of lung biopsy after resistance to first-line icotinib showed cylindrical morphology and formed glandular tubular structures. **(E–H)** Immunohistochemical staining was positive for NapsinA **(E)**, TTF-1 **(F)**, CK7 **(G)**, and CK20 **(H)**. **(I–L)** Immunohistochemical staining was positive for four MMR proteins. Magnification ×40 **(A–C, E–G, I–L)**; magnification ×200 **(D, H)**. NapsinA, novel aspartic proteinase of the pepsin family A; TTF-1, thyroid transcription factor-1; CK, cytokeratin; CDX2, caudal-type homeobox transcription factor 2; MMR, mismatch repair.

In February 2019, a 66-year-old man visited the First Affiliated Hospital of Guangxi Medical University due to a 2-month history of cough, sputum, and shortness of breath. The chest computed tomography (CT) scan showed a suspicious central right lung mass, with hilum of right lung, bilateral lung, and mediastinal lymph node metastasis; right-sided pleural effusion; and a small amount of pericardial effusion and atelectasis of the middle and lower lobes of the right lung (**Figure 1A**). The patient was a former heavy smoker with a smoking index of 20 packs/year. Cytological examination of pleural fluid revealed the diagnosis of malignant pleural effusion with the tumor cells positive staining for NapsinA and thyroid transcription factor-1 (TTF-1; **Figures 2A–C**). The patient was diagnosed with a metastatic lung adenocarcinoma (cT4N2M1, stage IV).

The patient immediately received one cycle of pemetrexed disodium (500 mg/m^2^) plus carboplatin (AUC 5) after diagnosis while waiting for the results of next-generation sequencing (NGS). Molecular analysis of pleural fluid by NGS based on a nine-gene panel was performed and showed the common *EGFR* exon 19 mutation (p.E746_A750del). The patient then received continuous oral icotinib (125 mg tid). After 1 month, CT showed a partial response (PR; **Figure 1B**). The clinical and morphological response was confirmed after 6 months since icotinib treatment. The patient continued icotinib beyond slow progression after 17 months of icotinib. The tumor continued to grow slowly over 3 months (**Figure 1C**), and a lung biopsy was obtained. The lung biopsy showed enteric differentiation components, which exceeded 50% (**Figure 2D**). Unfortunately, cell block at diagnosis was not available for further examination. Abdominal CT and colonoscopy examination revealed no evidence of gastrointestinal disease. Immunohistochemical (IHC) assays showed that the tumor was positive for TTF-1, NapsinA, CK7, and CDX2 (**Figures 2D–H**) and negative for programmed death ligand 1 (PD-L1; SP263 antibody), resulting in a diagnosis of an intestinal-type adenocarcinoma of the lung. IHC analysis for DNA mismatch repair proteins (MLH1, PMS2, MSH2, MSH6) revealed that the patient had proficient DNA mismatch repair (pMMR) positive for all microsatellite markers (**Figures 2I–L**). To identify actionable mutations, the tumor biopsy specimen was sequenced by capture-based next-generation DNA sequencing with a panel containing 1,021 cancer-related genes [(5) Beijing Geneplus Technology Co., Ltd.]. The mean effective depth of coverage of the sequence was 800×. A total of 35 somatic mutations were identified, including *EGFR* E19del, an acquired *EGFR* T790M mutation, and *MLH1* (c.332C>T, p.A111V mutation) with the highest variant allele fraction (VAF) of 45.2%, MSI-H, and high tumor mutation burden (TMB-H; 29.76 Muts/Mb) (**Supplementary Table 1**). No germline mutation in the coding region of *MSH2*, *MSH6*, *MLH1*, or *PMS2* was identified. The patient received osimertinib (80 mg daily), which allowed an improvement for 3 months until reassessment CT revealed pulmonary progression (**Figure 1D**), accompanied with increased hemoptysis and cough. From November 2020 to April 2021, he received two cycles of nedaplatin (180 mg)–pemetrexed (900 mg)–bevacizumab (600 mg) therapy, then four cycles of combination therapy with nedaplatin (180 mg) plus pemetrexed (900 mg), and one cycle of pemetrexed (900 mg) monotherapy. Pulmonary CT scans showed PR after first cycle of nedaplatin–pemetrexed–bevacizumab treatment and disease progression in May 2021 [progression-free survival (PFS) 6.5 months, **Figures 1E, F**]. Then, the patient received camrelizumab (anti-PD1, 200 mg) monotherapy (PFS 1 month) and camrelizumab (200 mg, q 3 weeks) plus pemetrexed (900 mg) therapy (**Figure 1G**). The patient’s condition worsened with multiple complications, including hemoptysis, pneumonia, and pulmonary fungal infections. He received supporting treatment and underwent another next-generation DNA sequencing test, which included 73 cancer-related genes (Beijing Geneplus Technology Co., Ltd.) with peripheral blood. The NGS results of plasma circulating tumor DNA showed loss of the T790M mutation with sustained presence of the *EGFR* exon 19 mutation (4.6% abundance, **Supplementary Table 1**). The patient started to receive gefitinib (250 mg/day) and everolimus (10 mg/day) therapy. He died 2 months later and had an overall survival of 30 months.

Descriptions of adjuvant therapies or systemic therapies are available in 34 PEAC patients derived from the literature and the case report reported above. The treatment regimens are summarized in **Supplementary Table 2** (6–19). Two cases with early-stage diseases received adjuvant chemotherapy with a regimen of unspecified drugs. As for patients with advanced disease, 22/34 received lung cancer-oriented chemotherapy, 2/34 received immunotherapy plus platinum-containing chemotherapy, 1/34 received icotinib without *EGFR* mutation and then nivolumab monotherapy, and 6/34 patients received colorectal cancer-oriented chemotherapy.

## Discussion

PEAC is a rare subtype of non-small-cell lung cancer (NSCLC), which was first reported by Tsao and Fraser as a case in 1991 (20). To date, over 200 cases have been reported (21). PEAC was officially defined as a rare variant of invasive lung adenocarcinoma by the WHO classification in 2015, with more than 50% of intestinal differentiation components, and the tumor cells should be positive for at least one immunohistochemical marker of enteric differentiation (1). The present case was first diagnosed as lung adenocarcinoma containing an *EGFR* mutation using tumor cells from pleural fluid. Then, the patient was diagnosed with adenocarcinoma with intestinal differentiation components from lung biopsy after resistance to first-line icotinib treatment. It may be difficult to diagnose PEAC using circulating tumor cells or a small sample of lung biopsy. Given the fact that PEAC is a mixed histological subtype of lung adenocarcinoma, the molecular differences between intestinal differentiation components and other non-intestinal differentiation components remain unknown. *EGFR* in-frame deletion of exon 19 (E19del) was reported in pulmonary enteric adenocarcinoma (1/10) (22). *EGFR* mutation may present in ordinary lung adenocarcinoma components, which decreased with the use of icotinib, while the intestinal differentiation components were insensitive to icotinib and increased during treatment. More molecular studies about the origin and intratumor heterogeneity of PEAC are needed in the future.

Owing to the rarity of disease, the molecular characteristics of PEAC have not been comprehensively determined. Several studies have described the genomic landscape of PEAC. High *KRAS* and *MMR* gene mutation rates and a low *EGFR* mutation rate were observed in PEAC (3, 4). The present case had a common *EGFR* E19del mutation and had a PFS of 17 months on first-line icotinib treatment. Genomic sequencing of tumor biopsy after icotinib resistance revealed *EGFR* T790M mutation. The patient received a third-generation EGFR tyrosine kinase inhibitor (TKI), osimertinib, and achieved a PFS of 3 months. This study is the first to describe *EGFR* tyrosine kinase inhibitors in a PEAC patient with an *EGFR*-sensitive mutation. The first- and second-line target therapies indicated that a PEAC patient with an *EGFR*-activating mutation could also benefit from *EGFR*-TKI, and an NGS test after first-line TKI resistance could guide subsequent therapy.

At present, the treatment plan of PEAC has not been fully studied in previous literature. The present treatment strategy for advanced PEAC is the same as that of primary lung adenocarcinoma, including chemotherapy and radiotherapy and/or targeted therapy. Immunotherapy might be a useful treatment option for PEAC because of its high frequency of MMR mutation (21). According to IMpower150, the atezolizumab (anti-PD-L1) plus bevacizumab plus chemotherapy regimen showed a progression-free and overall survival benefit when compared with the standard-of-care bevacizumab plus chemotherapy regimen in EGFRm-TKI progressed patients (23). The ORIENT-31 trial consistently demonstrated that combination therapy of sintilimab plus chemotherapy with or without bevacizumab biosimilar IBI305 significantly improved PFS compared with chemotherapy alone in EGFRm-TKI progressed patients (24). To date, only two case studies have demonstrated checkpoint inhibitor therapy in PEAC and exhibited controversial results. A recently published case showed that a metastatic PEAC patient with a *KRAS* G12C mutation suffered hyperprogressive disease after one cycle of first-line paclitaxel plus carboplatin, along with sindilizumab (25). However, another recently published study demonstrated that primary and metastatic lesions were effectively treated by pembrolizumab plus carboplatin and pemetrexed in a PEAC with a *KRAS* G12D mutation (26). The discrepancy of clinical benefits between the two PEAC cases receiving first-line chemoimmunotherapy might be explained by the different NGS panels used and metastatic status, and the latter case received palliative radiation for bone metastases. The effectiveness of combination therapy with a checkpoint inhibitor and chemotherapy in PEAC remained uncertain.

Our patient only received a bevacizumab plus chemotherapy regimen, without combination with a checkpoint inhibitor after progressing on treatment with icotinib and osimertinib. Then, the patient changed to ICI monotherapy or chemoimmunotherapy after progressing on treatment with bevacizumab plus chemotherapy, but he benefited poorly from those treatments. The presence of an *EGFR* mutation and *PTEN* and *JAK1* truncation mutations were negative predictors of immunotherapy (27–29) and might account for the treatment failure of the case. Accumulation of clinical experience in immunotherapy is necessary for better treatment of this rare lung cancer. Our case indicates poor benefit from immunotherapy for PEAC with an EGFR-sensitive mutation and MSI-H in later-line settings.

As we know, MSI-H is a more common molecular feature observed in colorectal and endometrial cancer compared with other solid tumors, while few studies concerned MSI status in lung cancer. Patients with MSI-H are more likely to benefit from immunotherapy across cancers (30). Polymerase chain reaction (PCR)-, IHC-, and NGS-based MSI analyses were commonly used in most clinical laboratories. However, it has been reported that approximately 5% of colorectal cancers that display retention of all four MMR proteins may indeed be MSI-H, possibly due to the heterogeneous expression of MMR proteins, or proteins emanating from abrogated MMR genes were still detected by IHC (31). The *MLH1* c.332C>T mutation, a germline pathogenic mutation reported in colorectal cancers (32), was detected in the lung biopsy of our case with the highest VAF. Missense mutations of MMR genes in formalin-fixed paraffin-embedded (FFPE) tumor tissues were also detected in colorectal cancer cases with pMMR and MSI-H (31). In lung cancer, 0.17% (2/1,153) and 0.5% (66/12,484) patients were reported to be MSI-H via IHC and the NGS-based method, respectively (33, 34). Recent studies showed all tumor tissue samples were microsatellite stable (MSS) in PEAC according to PCR- (17 cases) or IHC-based (8 cases) MSI analysis. The present case was a rare lung adenocarcinoma with enteric differentiation, pMMR, and MSI-H. Further studies in cases with somatic MMR gene mutations and MSI-H may help elucidate the phenomena.

In summary, this is the first case to describe an *EGFR*-mutated lung adenocarcinoma that had enteric differentiation components, *EGFR* T790M, and MSI-H after resistance to first-line icotinib and responded poorly to osimertinib and immunotherapy. Few reports to date have described the sequential treatment of PEAC with EGFR-TKIs and immunotherapy. The findings observed in this patient, including diagnoses, treatments, and the association between clinical outcomes and driver genes, may lead to future studies on the origin, diagnosis, and treatment of patients with PEAC.

## Data availability statement

The original contributions presented in the study are included in the article/**Supplementary Material**. Further inquiries can be directed to the corresponding authors.

## Ethics statement

The studies involving humans were approved by Ethics Committee of First Affiliated Hospital of Guangxi Medical University. The studies were conducted in accordance with the local legislation and institutional requirements. The participants provided their written informed consent to participate in this study. Written informed consent was obtained from the individual(s) for the publication of any potentially identifiable images or data included in this article.

## Author contributions

MY: Data curation, Investigation, Writing – original draft, Writing – review & editing. PY: Investigation, Writing – original draft, Writing – review & editing. ZH: Conceptualization, Writing – review & editing. JD: Conceptualization, Investigation, Writing – review & editing.

## References

[1] Travis WD Brambilla E Burke AP Marx A Nicholson AG . Introduction to the 2015 World Health Organization classification of tumors of the lung, pleura, thymus, and heart. J Thorac Oncol (2015) 10:1240–2. doi: 10.1097/JTO.0000000000000663 26291007

[2] Li H Cao W . Pulmonary enteric adenocarcinoma: a literature review. J Thorac Dis (2020) 12:3217–26. doi: 10.21037/jtd-19-4171 PMC7330779 32642243

[3] Chen M Liu P Yan F Xu S Jiang Q Pan J . Distinctive features of immunostaining and mutational load in primary pulmonary enteric adenocarcinoma: implications for differential diagnosis and immunotherapy. J Transl Med (2018) 16 81. doi: 10.1186/s12967-018-1449-z PMC5870381 29587865

[4] Zhang J Xiang C Han Y Teng H Li X Shao J . Differential diagnosis of pulmonary enteric adenocarcinoma and metastatic colorectal carcinoma with the assistance of next-generation sequencing and immunohistochemistry. J Cancer Res Clin Oncol (2019) 145:269–79. doi: 10.1007/s00432-018-2788-0 PMC11810349 30415301

[5] Ai X Cui J Zhang J Chen R Lin W Xie C . Clonal architecture of EGFR mutation predicts the efficacy of EGFR-tyrosine kinase inhibitors in advanced NSCLC: A prospective multicenter study (NCT03059641). Clin Cancer Res (2021) 27:704–12. doi: 10.1158/1078-0432.CCR-20-3063 33188140

[6] Li HC Schmidt L Greenson JK Chang AC Myers JL . Primary pulmonary adenocarcinoma with intestinal differentiation mimicking metastatic colorectal carcinoma: case report and review of literature. Am J Clin Pathol (2009) 131:129–33. doi: 10.1309/AJCPB04XWICTFERL 19095576

[7] Qureshi A Furrukh M . Enteric adenocarcinoma lung: a rare presentation in an Omani woman. BMJ Case Rep (2013) 2013. doi: 10.1136/bcr-2012-007667 PMC3603974 23355573

[8] Metro G Valtorta E Siggillino A Lauricella C Cenci M Ludovini V . Enteric-type adenocarcinoma of the lung harbouring a novel KRAS Q22K mutation with concomitant KRAS polysomy: a case report. Ecancermedicalscience (2015) 9:559. doi: 10.3332/ecancer.2015.559 26284123 PMC4531126

[9] Garajova I Funel N Fiorentino M Agostini V Ferracin M Negrini M . MicroRNA profiling of primary pulmonary enteric adenocarcinoma in members from the same family reveals some similarities to pancreatic adenocarcinoma-a step towards personalized therapy. Clin Epigenet (2015) 7:129. doi: 10.1186/s13148-015-0162-5 PMC4681170 26677401

[10] Lin LI Xu CW Zhang BO Liu RR Ge FJ Zhao CH . Clinicopathological observation of primary lung enteric adenocarcinoma and its response to chemotherapy: A case report and review of the literature. Exp Ther Med (2016) 11:201–7. doi: 10.3892/etm.2015.2864 PMC4726875 26889240

[11] El Hammoumi MM El Ochi R Kabiri el H . Primary lung adenocarcinoma with enteric morphology associated with primary colon adenocarcinoma. Arch Bronconeumol (2016) 52:221. doi: 10.1016/j.arbres.2015.05.012 26163114

[12] Shiina T Agatsuma H Saito G Toishi M Kondo R Yoshida K . A case of pulmonary enteric adenocarcinoma followed up as metastatic colorectal cancer. J Japanese Assoc Chest Surg (2016) 30:696–702. doi: 10.2995/jacsurg.30.696

[13] de Castria TB de Azevedo Souza MCL . Pulmonary adenocarcinoma with enteric differentiation: A distinctive histologic subtype. J Case Rep Images Oncol (2016) 2:23–7. doi: 10.5348/Z10-2016-15-CS-6

[14] Lin L Zhuang W Wang W Xu C Chen R Guan Y . Genetic mutations in lung enteric adenocarcinoma identified using next-generation sequencing. Int J Clin Exp Pathol (2017) 10:9583–90. PMC6966000 31966835

[15] Prakobkit R Churk-Nam Auyeung W Xu L Berry GJ . Pulmonary adenocarcinoma with enteric differentiation presenting with bronchorrhea. J Thorac Oncol (2017) 12:e120–3. doi: 10.1016/j.jtho.2017.04.005 28748820

[16] Miyaoka M Hatanaka K Iwazaki M Nakamura N . CK7/CK20 double-negative pulmonary enteric adenocarcinoma with histopathological evaluation of transformation zone between enteric adenocarcinoma and conventional pulmonary adenocarcinoma. Int J Surg Pathol (2018) 26:464–8. doi: 10.1177/1066896918756737 29411669

[17] Todisco A Interno V Stucci LS Ostuni C Lovero D D’Oronzo S . Cutaneous metastasis as a primary presentation of a pulmonary enteric adenocarcinoma. Int J Biol Markers (2019) 34:421–6. doi: 10.1177/1724600819877190 31556336

[18] Tu LF Sheng LY Zhou JY Wang XF Wang YH Shen Q . Diagnosis and treatment of primary pulmonary enteric adenocarcinoma: Report of Six cases. World J Clin cases (2021) 9:9236–43. doi: 10.12998/wjcc.v9.i30.9236 PMC8567515 34786410

[19] Fassi E Mandruzzato M Zamparini M Bianchi S Petrelli F Baggi A . Clinical presentation and outcome of patients with enteric-type adenocarcinoma of the lung: A pooled analysis of published cases. Lung Cancer (2023) 179:107176. doi: 10.1016/j.lungcan.2023.107176 37015149

[20] Tsao MS Fraser RS . Primary pulmonary adenocarcinoma with enteric differentiation. Cancer (1991) 68:1754–7. doi: 10.1002/1097-0142(19911015)68:8<1754::aid-cncr2820680818>3.0.co;2-e 1913519

[21] Gong J Fan Y Lu H . Pulmonary enteric adenocarcinoma. Transl Oncol (2021) 14:101123. doi: 10.1016/j.tranon.2021.101123 34000642 PMC8141771

[22] Xie M Chen D Li Y Liu X Kuang D Li X . Genetic mutation profiles and immune microenvironment analysis of pulmonary enteric adenocarcinoma. Diagn Pathol (2022) 17:30. doi: 10.1186/s13000-022-01206-7 35172862 PMC8849039

[23] Reck M Mok TSK Nishio M Jotte RM Cappuzzo F Orlandi F . Atezolizumab plus bevacizumab and chemotherapy in non-small-cell lung cancer (IMpower150): key subgroup analyses of patients with EGFR mutations or baseline liver metastases in a randomised, open-label phase 3 trial. Lancet Respir Med (2019) 7:387–401. doi: 10.1016/S2213-2600(19)30084-0 30922878

[24] Lu S Wu L Jian H Cheng Y Wang Q Fang J . Sintilimab plus chemotherapy for patients with EGFR-mutated non-squamous non-small-cell lung cancer with disease progression after EGFR tyrosine-kinase inhibitor therapy (ORIENT-31): second interim analysis from a double-blind, randomised, placebo-controlled, phase 3 trial. Lancet Respir Med (2023) 11:624–36. doi: 10.1016/S2213-2600(23)00135-2 37156249

[25] Hu CH Shi S Dong W Xiao L Zang H Wu F . Hyperprogressive disease after immunotherapy: A case report of pulmonary enteric adenocarcinoma. Front Oncol (2022) 12:799549. doi: 10.3389/fonc.2022.799549 35321429 PMC8937032

[26] Teranishi S Sugimoto C Nagayama H Segawa W Miyasaka A Hiro S . Combination of pembrolizumab with platinum-containing chemotherapy for pulmonary enteric adenocarcinoma. Cancer Diagn Progn (2022) 2:253–7. doi: 10.21873/cdp.10102 PMC8962809 35399182

[27] Marabelle A Aspeslagh S Postel-Vinay S Soria JC . JAK mutations as escape mechanisms to anti-PD-1 therapy. Cancer Discovery (2017) 7:128–30. doi: 10.1158/2159-8290.CD-16-1439 28167612

[28] Peng W Chen JQ Liu C Malu S Creasy C Tetzlaff MT . Loss of PTEN promotes resistance to T cell-mediated immunotherapy. Cancer Discovery (2016) 6:202–16. doi: 10.1158/2159-8290.CD-15-0283 PMC4744499 26645196

[29] Rizvi H Sanchez-Vega F La K Chatila W Jonsson P Halpenny D . Molecular determinants of response to anti-programmed cell death (PD)-1 and anti-programmed death-ligand 1 (PD-L1) blockade in patients with non-small-cell lung cancer profiled with targeted next-generation sequencing. J Clin Oncol (2018) 36:633–41. doi: 10.1200/JCO.2017.75.3384 PMC6075848 29337640

[30] Marabelle A Le DT Ascierto PA Di Giacomo AM De Jesus-Acosta A Delord JP . Efficacy of pembrolizumab in patients with noncolorectal high microsatellite instability/mismatch repair-deficient cancer: results from the phase II KEYNOTE-158 study. J Clin Oncol (2020) 38:1–10. doi: 10.1200/JCO.19.02105 31682550 PMC8184060

[31] McCarthy AJ Capo-Chichi JM Spence T Grenier S Stockley T Kamel-Reid S . Heterogenous loss of mismatch repair (MMR) protein expression: a challenge for immunohistochemical interpretation and microsatellite instability (MSI) evaluation. J Pathol Clin Res (2019) 5:115–29. doi: 10.1002/cjp2.120 PMC6463865 30387329

[32] Kurzawski G Suchy J Lener M Klujszo-Grabowska E Kladny J Safranow K . Germline MSH2 and MLH1 mutational spectrum including large rearrangements in HNPCC families from Poland (update study). Clin Genet (2006) 69:40–7. doi: 10.1111/j.1399-0004.2006.00550.x 16451135

[33] Yanagawa N Yamada N Sugimoto R Osakabe M Uesugi N Shiono S . The frequency of DNA mismatch repair deficiency is very low in surgically resected lung carcinoma. Front Oncol (2021) 11:752005. doi: 10.3389/fonc.2021.752005 34692533 PMC8527876

[34] Tian J Wang H Lu C Liu L Zhang X Xie Y . Genomic characteristics and prognosis of lung cancer patients with MSI-H: A cohort study. Lung Cancer (2023) 181:107255. doi: 10.1016/j.lungcan.2023.107255 37244039

