# Supplementary material for: Case report: Target and immunotherapy of a lung adenocarcinoma with enteric differentiation, EGFR mutation, and high microsatellite instability
Source: Front Immunol. 2024 Jan 25;14:1266304. doi: 10.3389/fimmu.2023.1266304 (PMC10850318; doi:10.3389/fimmu.2023.1266304)
Supplement: Supplementary file 2 [file Table_2.docx]

**Supplementary Table 2**. Treatment regimens of reported cases of pulmonary adenocarcinoma with enteric differentiation.

| Author/Year | Type of Study | Number of patients | Disease Stage | Reference | Adjuvant therapy | First-line | Second-line | Other therapy |
| --- | --- | --- | --- | --- | --- | --- | --- | --- |
| Li et al./2009 | Case Report/Review | 1 | IIB | (6) | Chemotherapy | - |  |  |
| Qureshi et al./2013 | Case Report | 1 | IV | (7) | - | Pemetrexed + carboplatin |  |  |
| Metro et al./2015 | Case Report | 1 | IV | (8) | - | Single-agent gemcitabine | Pemetrexed |  |
| Garajová et al./2015 | Case Report | 2 | IIB | (9) | - | XELOX (capecitabine + oxaliplatin) | Carboplatin + Pemetrexed | Docetaxel |
|  |  |  | IB |  | - | Carboplatin + pemetrexed, followed by pemetrexed alone |  |  |
| Lin el al./2016 | Case Report/Review | 1 | IV | (10) | - | XELOX chemotherapy regimen | TP regimen | FOLFIRI regimen |
| El Hammoumi et al./2016 | Case Report | 1 | IB | (11) | - | Chemotherapy |  |  |
| Shiina et al./2016 | Case Report/Review | 1 | IIIA | (12) | - | Chemotherapy |  |  |
| Castria et al./2016 | Case Series | 5 | IB: 1 pts | (13) | - | - |  |  |
|  |  |  | IV: 4 pts |  | - | 3 cases received mFOLFOX6 or FOLFIRI regimens, one received chemotherapy |  |  |
| Lin et al./2017 | Case Series | 11 | IIIb-IV | (14) | - | Case 1 received icotinib for more than 1.5 months without EGFR mutation and then nivolumab for 9.5 months, other patients received chemotherapy. |  |  |
| Prakobkit et al./2017 | Case Report | 1 | IV | (15) | - | Carboplatin + paclitaxel |  |  |
| Miyaoka et al./2018 | Case Report | 1 | IIA | (16) | chemotherapy | - |  |  |
| Todisco et al./2019 | Case Report | 1 | IV | (17) | - | Pemetrexed + Cisplatin |  |  |
| Tu et al./2021 | Case Report | 6 | IA-II: 2pts | (18) | - | - |  |  |
|  |  |  | IV: 4pts |  | - | 1 case received chemotherapy and bevacizumab, two cases received pemetrexed + carboplatin | Pemetrexed + carboplatin |  |
| Teranishi et al./2022 | Case Report | 1 | IV | (19) | - | Pembrolizumab plus platinum-containing chemotherapy. |  |  |
| Hu et al./2022 | Case Report | 1 | IV | (20) | - | Chemotherapy plus sindilizumab immunotherapy |  |  |
| Fassi et al./2023 | Case Report/Review | 1 | IV | (21) | - | Oxaliplatin, leucovorin and fluorouracil (FOLFOX regimen) | FOLFIRI | Nivolumab |
| Present Case | Case Report | 1 | IV |  | - | Icotinib | Osimertinib | Chemotherapy± bevacizumab, camrelizumab± chemotherapy |
